# Straw return combined with single-season phosphorus application for rapeseed enhances the annual productivity and phosphorus use efficiency under rapeseed–rice rotation

**DOI:** 10.3389/fpls.2026.1768595

**Published:** 2026-03-17

**Authors:** Keyuan Zhang, Xiaotian Jiang, Lubing Jia, Jianming Ding, Ran Wang, Jun Deng, Erluo Yang, Rongping Zhang, Yan Lan, Yungao Hu, Peng Ma

**Affiliations:** College of Life Sciences and Agri-forestry, Southwest University of Science and Technology, Mianyang, Sichuan, China

**Keywords:** phosphorus utilization, phosphorus-soluble bacteria, rapeseed-rice rotation, straw return, yield

## Abstract

**Introduction:**

The rapeseed–rice rotation system involves high phosphorus application rates in both seasons, with low phosphorus use efficiency and a large amount of straw produced. However, the synergistic mechanism of phosphorus fertilizer management and straw return in this system remains unclear.

**Methods:**

A two-factor split-plot experiment was designed to systematically investigate the effects of straw incorporation and seasonal phosphorus (P) reduction on annual productivity, P-use efficiency, soil fertility, and microbial community structure in a rapeseed-rice rotation system in 2022-2024. The main plot was no straw return (S1) and straw return (S2) for both the rapeseed and rice seasons. The subplot was set with three P application modes: no P application throughout the year (P1), P application throughout the year (P2), and P application only during the rapeseed season (P3) .

**Results:**

S2 significantly enhanced annual yield and P-use efficiency of the rapeseed-rice rotation system, with the best results observed under treatment P3. Under treatment S2, the P fertilizer uptake and utilization rate increased by 22.28%, the partial factor productivity of P fertilizer improved by 19.4%, and the agronomic efficiency of P fertilizer increased by 67.52% compared to that under treatment S1. The P3 treatment further improved P-use efficiency, with the P fertilizer uptake and utilization rate being 92.51% higher than that of the P2 treatment. Additionally, the S2P3 treatment significantly increased rice root morphology and ATPase activity, soil organic matter content, the total P contents and the relative abundance of soil P-soluble bacteria in the soil.

**Discussion:**

Straw return combined with rapeseed single-season P application significantly increased the annual productivity of the rapeseed-rice rotation system, improved the ATPase of rice, and increased the total P and Olsen-P contents in the soil through straw decomposition and the activity of soil P-soluble bacteria. This significantly improved P-uptake efficiency, achieved P reduction and increased P-use efficiency.

## 1 Introduction

Crop rotation can effectively enhance arable land-use efficiency, and therefore, has been extensively promoted across multiple regions. Compared with rice-wheat rotation and fallowing in winter, rapeseed-rice rotation not only effectively increases rice yield, but also improves soil properties during the cultivation process (Weiser et al., 2018). The application of P fertilizer is substantial in both seasons in the rapeseed-rice rotation system, and excessive P fertilizer input leads to the accumulation of large amounts of unavailable P in the soil. This not only wastes the limited phosphate rock resources but also restricts the sustainable development of agriculture. Data analysis indicates that the annual P fertilizer input in China’s farmland accounts for more than 30% of the global total (Zhou et al., 2017). However, owing to the strong fixation of P in soil, the utilization rate of P fertilizer during the application stage is only 10–25% (Du et al., 2021). With the continuous improvement in agricultural production, crop yields have been increasing annually, as has the amount of straw produced. China has the highest straw production worldwide. The total amount of straw resources has reached one billion tons, accounting for 20–30% of the global straw production (Shun et al., 2024). Straw return is a major measure for the comprehensive utilization of agricultural waste resources, a primary way to improve soil fertility and structure in farmland, and an important means to replace chemical fertilizers with organic materials, thereby reducing fertilizer application and improving use efficiency (Tian et al., 2019; Dandan et al., 2022; Zhang et al., 2023a).

Mineral nutrients released from the decomposition of straw directly supply the needs of crops and regulates crop physiological metabolism by improving the rhizosphere microenvironment. Straw return can increase the annual yield in a rapeseed-rice rotation system and significantly improve the P fertilizer production efficiency of rice and rapeseed, the average agronomic efficiency of P in both rice and rapeseed seasons, and the annual cumulative utilization rate of P (Wang et al., 2020). The reason is that straw return can increase the input of soil nutrients by adding plant stem and leaf residues to the soil, which serve as a source of available P. Generally, crops utilize 6–15% of the total P in straw (Espinosa et al., 2017). In addition, straw return can significantly promote the mineralization of soil organic P and effectively regulate the components of soil P (Wang et al., 2025b). In this case, the additional application of P fertilizer may lead to an excess of P in the soil. Therefore, when the straw return amount is sufficient, the application of P fertilizer can be appropriately reduced to improve its utilization rate of P fertilizer.

Phosphorus is included in the composition of important organic compounds in plants and plays a crucial role in adenosine triphosphate (ATP) synthesis. It is also involved in other physiological and biochemical processes in plants and regulates their physiological metabolism, growth, and development (Li et al., 2005). However, P exists in various forms in the soil, and different forms of P differ significantly in their availability for crop growth. Organic phosphates are mainly converted into available P through mineralization, which can then be absorbed by crops, whereas inorganic phosphates are transformed into soluble inorganic P through solubilization by microorganisms (Alan and Richard, 2011). Soil microorganisms are important components of agricultural ecosystems and play a key role in soil P cycling and mineralization of soil organic P. They can effectively promote the transformation of soil organic matter (SOM), enhance soil fertility, and facilitate crop uptake of soil nutrients (Tian et al., 2021). Microorganisms release P through biomass turnover, thereby increasing the available P content in the soil (Heuck et al., 2015). Studies have confirmed that the application of external P can significantly increase the content of available P in the soil, thereby affecting the composition of soil microbial communities, increasing the abundance of beneficial microorganisms, and making them the dominant bacterial groups (Tian et al., 2021).

In the paddy-upland rotation, the seasonal dry-wet alternation of soil affect the soil redox process and changed the form and availability of P (Fan et al., 2008). The specific performance was that the soil dissolved oxygen increased and decreased water content in the dry season, resulting in the combination of PO_4_^3-^ and metal ions to form insoluble phosphate compounds (Lv et al., 1995). Under flooding conditions, the reduction of Fe^3+^ ion led to the gradual dissolution of insoluble phosphates in the soil, thereby increasing the supply of available P in the soil (Flessa and Fischer, 1992). Cruciferous crops such as rapeseed have a high demand for phosphate fertilizer, while cereal crops such as rice have a relatively low demand for phosphate fertilizer. Combined with the dynamic changes and residual effects of soil phosphorus availability, it is generally considered that the application of phosphate fertilizer is based on the management strategy of dry-season heavy, wet-season light (Zhu et al., 2016; Yuan et al., 2017). Studies have confirmed that allocating a large amount of P fertilizer during the dry season in paddy-upland rotation can increase crop yield (Fan et al., 2008). Previous studies have found that in the rapeseed-rice rotation system, reducing the annual application of P and applying all phosphorus in the rice season cannot meet the high-yield phosphorus demand of rapeseed. However, applying all phosphorus in the rapeseed season can make the phosphorus absorption efficiency in the rice season higher than the traditional fertilization method (Zhang et al., 2023b).

Currently, the research on the combined mechanism of straw return and reduced P fertilizer application remains unclear, especially due to lack of quantitative evidence on the impact on the rapeseed-rice rotation system. Therefore, in this study, we aimed to clarify the underlying mechanisms of P reduction and efficiency enhancement under straw return. The specific objectives were as follow: (1) to explore whether the residual P in the rape season combined with straw return can meet the growth needs of rice season; (2) to explore the interaction of rice root-soil-microorganism under different straw return and phosphate fertilizer conditions; (3) assessing the stability of annual crop yield of rapeseed-rice under the condition of straw return combined with rapeseed single-season P application. The findings of this study will provide a management direction for the reduction and efficiency of phosphorus fertilizer under rapeseed-rice rotation.

## 2 Materials and methods

### 2.1 Site description, experimental design and fertilizer treatments

The located experiment was conducted at the rice experimental base of the Southwest University of Science and Technology in Mianyang City, Sichuan Province (104.67°E, 31.31°N). The experiment began in the rice season of 2017 in a rapeseed-rice rotation system, the data in this paper were mainly presented from 2022 to 2024. The soil in the experimental field was fluvo-aquic, a typical soil type in this area. The meteorological data during the experiment are shown in **Supplementary Figure 1**. The basic physicochemical properties of the soil in the 0–30 cm soil layer before the positioning test were as follows: organic matter content, 21.5 g·kg^-1^; total N content, 1.61 g·kg^-1^; alkali-hydrolyzed N content, 80.3 mg·kg^-1^; total P content, 581 mg·kg^-1^; available P content, 17.52 mg·kg^-1^; total K content, 12.8 g·kg^-1^; available K content, 89.55 mg·kg^-1^; and pH, 6.9.

The experiment used a two-factor split-plot design, and two straw treatments were set up in the main plot: no straw return during the rapeseed-rice season (S1) and straw return during the rapeseed-rice season (S2), and three P application modes were set up in the sub-area: rapeseed-rice annual production with no P application (P1), rapeseed-rice annual production with P application (P2), and single-season P application during the rapeseed season (P3). Each treatment was replicated three times, and the area of each subplot was 10 m². Nitrogen (180 kg·hm^-2^), K (150 kg·hm^-2^), and P (120 kg·hm^-2^) were applied during the rapeseed season, with N fertilizer applied as a base and overwintering fertilizer at a ratio of 5:5. During the rice season N (150 kg·hm^-2^), K (150 kg·hm^-2^), and P (90 kg·hm^-2^) were applied. Nitrogen fertilizer was applied as a base, tillering, and panicle fertilizer at a ratio of 4:3:3. Phosphorus and K fertilizers were applied as basal fertilizers in both seasons and applied one day before transplantation. The tested fertilizers were urea (N, 46%), potassium chloride (K_2_O, 60%) and calcium superphosphate (P_2_O_5_, 12%).

In the plots with no straw return, the upper part of the previous straw was removed. In the plots with straw return, the previous straw was cut into small sections of 5–7 cm, and the straw was converted into a 15 cm soil layer using a rotary tiller. The winter rapeseed variety Guohaoyou 7 was subjected to the seedling-transplanting method. The seedlings were reared in early September each year, and transplanting was performed in mid to late October. The plant spacing was 35 cm × 25 cm, and plants were harvested in May the following year. The rice variety Taiyou 808 was grown in mid-April every year and transplanted in June. The plant spacing was 36 cm × 18 cm, and the plants were harvested in late September.

### 2.2 Sampling and measurements

#### 2.2.1 Annual yield, dry matter, and P accumulation of rapeseed-rice

Three representative plants were selected from each plot according to the average number of tillers or branches at the maximum tillering, full heading and maturity stage of rice and the budding and maturity stage of rapeseed. The plants were dried, weighed, and ground to determine dry matter content. For each sample, 0.2 g was weighed and digested with H_2_SO_4_–H_2_O_2_. Plant P content was determined using the molybdenum-antimony resistance colorimetric method. And calculated the phosphorus uptake in plants (Equation 1), annual P uptake (Equation 2), annual P fertilizer recovery efficiency (Equation 3), partial factor productivity of P fertilizer (Equation 4), agronomic efficiency of P fertilizer (Equation 5). Five representative plants at the mature stage were collected from each subplot for seed quality analysis, and 15 plants were used to determine the yield.

#### 2.2.2 ATPase activity

At the heading stage of rice, the tillers of rice heading on the same day were marked. After rice heading, the flag leaves were sampled every 7 days for four consecutive times, and the ATPase activity of rice was determined using a Ca^++^-Mg^++^-ATPase activity assay kit (Solarbio).

#### 2.2.3 Soil nutrient content and microbial community

Soil samples were collected 30 days after rice transplantation, at rice maturity, and during the rapeseed bolting stage, before rice transplantation (after rapeseed harvest). Five-point sampling method was used in each plot. Soil samples with depths of 0–15 cm and 16–30 cm were taken by tubular soil auger and mixed evenly. A part of the soil samples were stored at 4 °C for the determination of soil microbial diversity. The remaining samples were air-dried, ground and sieved for the subsequent determination of soil organic matter and phosphorus components. The total P in the soil was determined by digestion with CuSO_4_–H_2_SO_4_ and measured using the molybdenum-antimony resistance colorimetric method. Olsen-P was determined using the NaHCO_3_ extraction method, and total organic matter in the soil was measured using the dilution heat method.

#### 2.2.4 Soil microbial community

Fresh soil samples collected 30 days after rice transplantation were sent to the Biomarker Technologies Corporation in Beijing for 16sRNA sequencing. Microbial diversity study is mainly processed on Illumina Novaseq platform by generating PE reads targeted regions. These short reads are assembled, clustered and de-noised to obtain tags, which can be used for species annotation and abundance analysis.

### 2.3 Data analysis

(1) $$\begin{array}{l} \text{Phosphorus uptake in plants }(\text{kg}\cdot {\text{hm}}^{-2})=\\ \text{Biomass}\times \text{P content in plants} \end{array}$$

(2) $$\begin{array}{l} \text{Annual P uptake }(\text{kg}\cdot {\text{hm}}^{-2})\\ =\text{P uptake in aboveground parts during the rapeseed season}\\ +\text{P uptake in aboveground parts during the rice season} \end{array}$$

(3) $$\begin{array}{l} \text{Annual P fertilizer recovery efficiency }(\%)\\ \frac{\left(\text{P uptake in the fertilized treatment }-\text{ P uptake in the unfertilized treatment}\right)}{\text{Annual P fertilizer application rate}}\times 100 \end{array}$$

(4) $$\begin{array}{l} \text{Partial factor productivity of P fertilizer }(\text{kg}\cdot {\text{kg}}^{-1})=\\ \text{Grain yield in the fertilized area}/\text{P fertilizer application rate} \end{array}$$

(5) $$\begin{array}{l} \text{Agronomic efficiency of P fertilizer }(\text{kg}\cdot {\text{kg}}^{-1})=\\ \frac{\left(\text{Grain yield in the fertilized area }-\text{ Grain yield in the unfertilized area}\right)}{\text{P fertilizer application rate}} \end{array}$$

All experimental data were collated using Microsoft Excel software, SPSS (version 26.0) was used to analyze the variance according to LSD at a significance level of *p* < 0.05, and plotted using Origin (version 2021) software.

## 3 Results

### 3.1 Rapeseed-rice dry matter weight and yield

**Figure 1:**
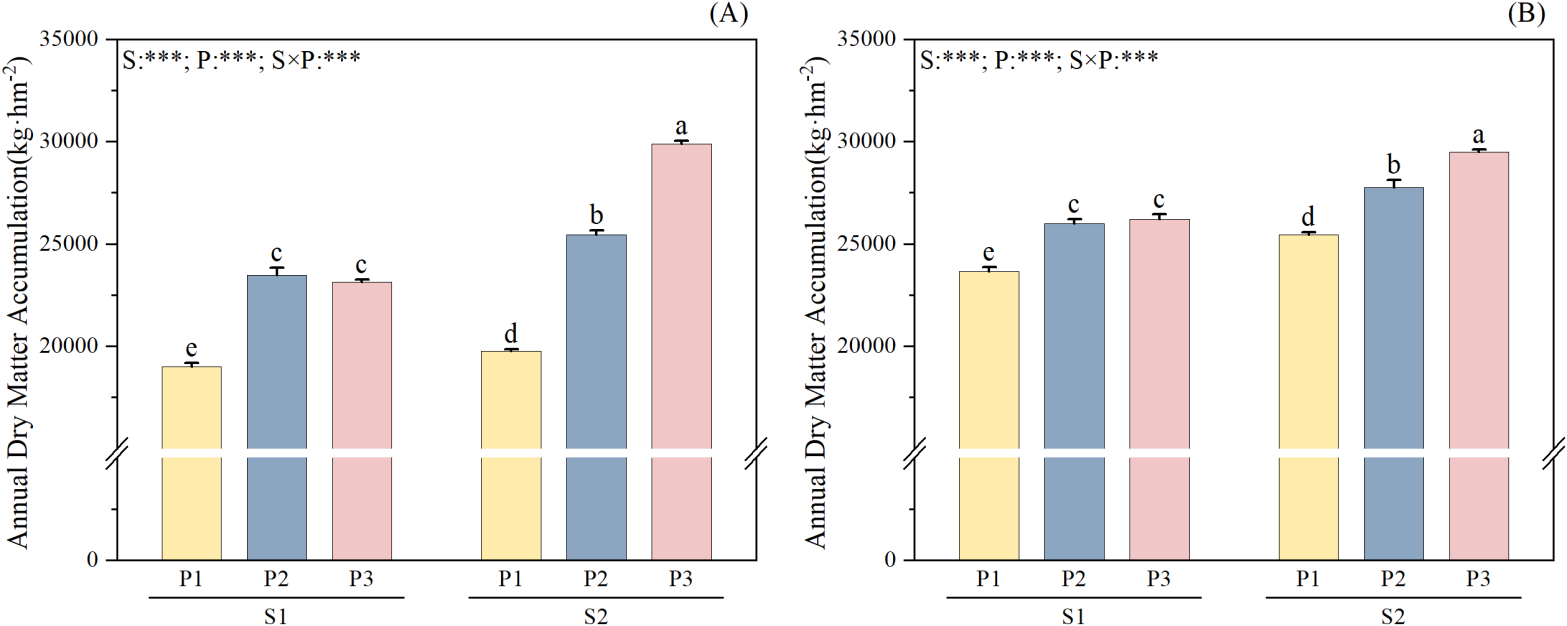
Effects of straw return and phosphate fertilizer treatment on annual dry matter accumulation. **(A)** 2022-2023, **(B)** 2023-2024, the results of ANOVA between different treatments in the lowercase alphabet. S, P, S × P are the interaction between straw return treatment, phosphate fertilizer treatment, straw return and phosphate fertilizer treatment, respectively. The asterisk indicates significant difference, ***P < 0.001. The error bars represent the ± standard deviation of the mean (n = 3).

The interaction between straw return treatment (S), phosphate fertilizer treatment (P), straw return and phosphate fertilizer treatment (S×P) had extremely significant effects on the annual dry matter accumulation rapeseed-rice rotation (*P* < 0.001). Under the same P fertilizer treatment, straw return increased the annual dry matter accumulation of rapeseed-rice rotation compared to that under no straw return, with consistent interannual performance (**Figure 1**). The annual dry matter accumulation under S2 was 4.06-29.10% higher than that under S1 in each P fertilizer treatment (P1–P3). In the rice season, the dry matter accumulation under S2 was 4.48-13.10%, 5.00-10.30%, and 3.30-11.74% higher than that under S1 (**Supplementary Figure 2**). In the rapeseed season, the dry matter accumulation under S2 was 8.69-40.02%, 12.50-31.95%, and 45.71-68.73% higher than that under S1 (**Supplementary Figure 3**). Under the same straw treatment, the dry matter accumulation of rice in each period followed the order of P3 > P2 >P1. The dry matter accumulation of rapeseed showed a trend of P2 > P3 > P1 under S1, under S2 a trend of P3 > P2 > P1 was observed. The dry matter accumulation of rapeseed-rice in two seasons was the highest in S2P3 treatment. The annual dry matter accumulation in S2P3 was 6.28-57.27% higher than that in the other treatments.

As shown in **Table 1**, in the two-year field experiment, straw return and rapeseed single-season P application significantly increased rice yield (*P* < 0.05). Under the same phosphate fertilizer treatment, the rice yield showed a consistent trend of S2 > S1, under the same straw treatment, the rice yield followed the order of P3 > P2 >P1, S2P3 was 7.74–37.39% higher than that in the other treatments. Under all P fertilizer treatments, the panicle number of rice followed the trend of S2 > S1, and the panicle number of rice under S2 was 0.78–9.89% higher than that under S1. The grain filling of rice under S2 increased significantly in the P3 treatment, which was 0.27–6.5% higher than that in the other treatments. Under the same straw return conditions, the panicle number and grain number per panicle of rice were the highest in the P3 treatment. The panicle number and grain number per panicle in P3 was 8.33–10.26% and 0.69–18.56% higher than that in other treatments, respectively. Straw return mainly affects the number of effective panicles of rice, and thus, affects rice yield, whereas P fertilizer affects the number of effective panicles of rice and the number of grains per panicle.

**Table 1:** Effects of straw return and phosphate fertilizer management on rice yield and yield traits.

Year	Treatment		Panicles (×10^4^·hm^-^^2^)	Spikelet per panicle	Grain filling (%)	1000-grain weight(g)	Grain yield (kg·hm^-2^)
2022	S1	P1	187.24c	172.25d	75.62d	26.49c	6138.61e
		P2	188.52c	194.88b	77.79bc	26.33d	6780.62d
		P3	206.21a	204.22a	78.79b	26.68ab	7808.93b
		Mean	193.99	183.78	77.4	26.50	6909.39
	S2	P1	205.76a	182.90c	77.06c	26.77a	7565.43c
		P2	194.88b	206.17a	77.24c	26.67ab	7827.89b
		P3	207.83a	207.60a	82.08a	26.59bc	8433.77a
		Mean	202.82	198.89	78.79	26.68	7942.36
2023	S1	P1	170.00d	210.27b	90.18b	27.62a	7718.48e
		P2	170.49d	214.76ab	92.40a	27.28ab	7863.41de
		P3	186.17b	218.92a	89.22b	26.77b	8453.87c
		Mean	175.55	214.65	90.60	27.22	8011.92
	S2	P1	179.05bc	204.22c	89.83b	27.63a	7950.53d
		P2	175.93c	214.63ab	91.76a	27.35ab	8792.18b
		P3	193.98a	216.94a	92.01a	27.42ab	9618.54a
		Mean	182.99	211.76	91.20	27.47	8787.08
F Value	Y		72.217**	2831.113**	461.927**	280.684**	914.2**
	S		5.551*	17.275**	83.408**	7.334*	788.238**
	P		3.382	33.305**	122.849**	113.339**	499.728**
	Y×S		0.135	2.733	0.618	24.39**	16.026**
	Y×P		3.077	30.84**	2.791	26.083**	0.968
	S×P		0.186	19.84**	10.228**	1.48	2.042
	Y×S×P		3.631*	1.138	6.445**	2.519	61.622**

Different lowercase letters in the same column represent ANOVA results between different treatments in the same year; Y, S and P in the F value are year, straw return treatment and phosphate fertilizer treatment, respectively. Y × S, Y × P, S × P and Y × S × P are the interaction between year, straw return treatment and phosphate fertilizer treatment, respectively. The asterisk indicates significant difference, *P < 0.05, **P < 0.01.

The interaction between S, P, and S×P had extremely significant effects on the rapeseed yield (*P* < 0.05). Under the same phosphate fertilizer treatment, the rapeseed yield showed a consistent trend of S2 > S1 over two years (**Table 2**). The yield of rapeseed under S2 was 15.97–23.19%, 4.57–22.01%, and 34.71–45.63% higher than that under S1 in each phosphate fertilizer treatment. The rapeseed yield under S1 was the highest in the P2 treatment, under S2 was the highest in the P3 treatment, which was 5.24–112.4% higher than other treatments. For yield components, the number of siliques per plant and number of seeds per silique of rapeseed under each phosphate fertilizer treatment showed a trend of S2 > S1, with that under S2 being 0.29–37.98% and 5.73–22.6% higher than S1, respectively. Under straw return, the P3 treatment had the highest siliques per plant, which was 1.71–78% higher than that in the other treatments. The number of seeds per silique was the highest in P3 treatment (2.59–25.79% higher than the other treatments) under both S1 and S2. Straw return can increase rapeseed yield by altering the number of siliques per plant and number of seeds per silique. The effect of the P3 treatment appeared to be the best.

**Table 2:** Effects of straw return and phosphate fertilizer management on yield and yield traits of rapeseed.

Year	Treatment		Siliques per plant	Seeds per silique	1000-seed weight(g)	Yield (kg·hm^-2^)
2023	S1	P1	187.00d	13.65c	3.44b	1032.88f
		P2	349.50a	16.20b	3.16e	2104.97c
		P3	258.00b	17.16b	3.57a	1855.80d
		Mean	264.83	15.67	3.39	1664.55
	S2	P1	200.00c	16.73b	3.23d	1272.41e
		P2	350.33a	19.13a	3.26d	2568.19b
		P3	356.00a	19.62a	3.35c	2702.64a
		Mean	302.11	18.49	3.28	2181.08
2024	S1	P1	417.00e	13.74c	3.06ab	2061.17e
		P2	656.33b	13.73c	3.01bc	3184.91c
		P3	574.00c	14.46bc	3.18a	3101.92c
		Mean	549.11	14.31	3.08	2782.67
	S2	P1	444.33d	15.05b	3.04b	2390.26d
		P2	671.67b	14.62b	2.89c	3330.31b
		P3	745.33a	16.17a	2.95bc	4187.63a
		Mean	620.44	15.28	2.96	3302.73
F Value	Y		325.742**	12255.219**	119.882**	3062.161**
	S		43.238**	380.341**	84.98**	653.863**
	P		38.264**	2015.237**	28.417**	1560.451**
	Y×S		0.18	44.043**	11.49**	0
	Y×P		5.105*	161.165**	13.996**	40.759**
	S×P		12.716**	209.19**	0.139	121.268**
	Y×S×P		11.719**	15.36**	0.769	16.511**

Different lowercase letters in the same column represent ANOVA results between different treatments in the same year; Y, S and P in the F value are year, straw return treatment and phosphate fertilizer treatment, respectively. Y × S, Y × P, S × P and Y × S × P are the interaction between year, straw return treatment and phosphate fertilizer treatment, respectively. The asterisk indicates significant difference, *P < 0.05, **P < 0.01.

### 3.2 ATPase activity of rice

**Figure 2:**
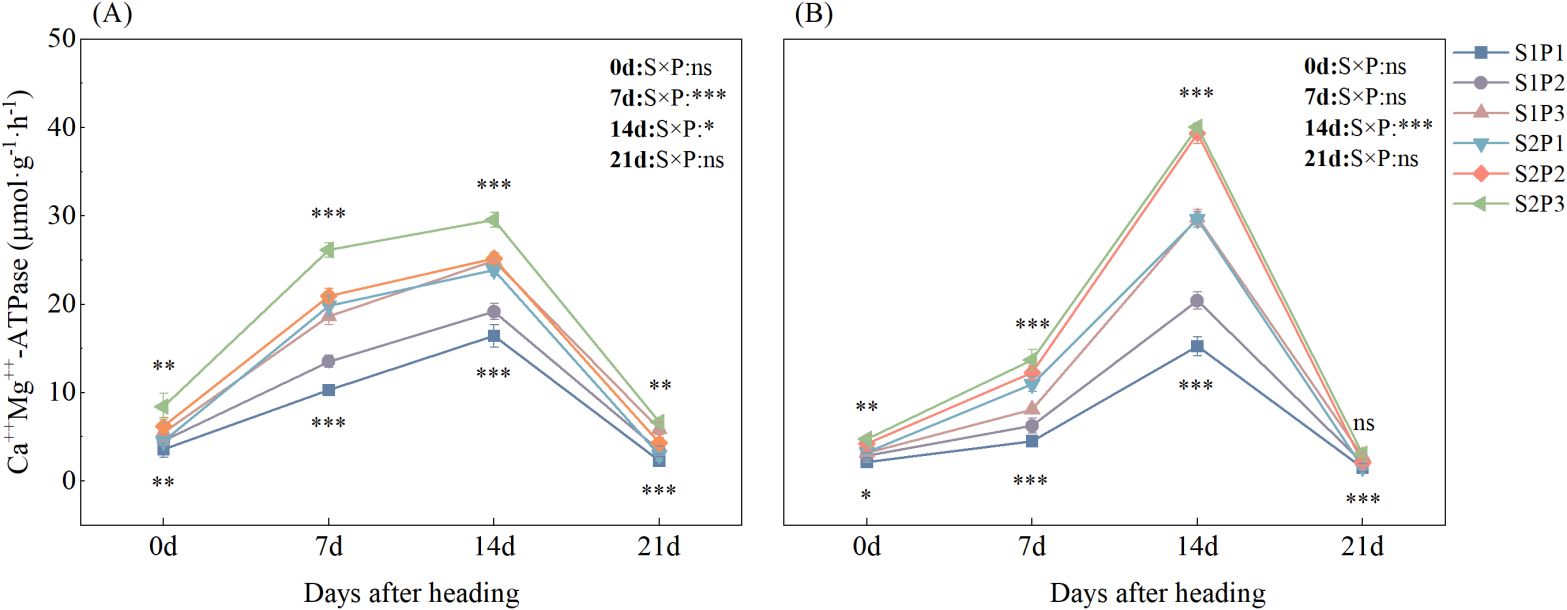
Effects of straw return and phosphorus fertilizer treatments on rice ATPase activity after heading in 2022 **(A)** and 2023 **(B)**. The annotations above the lines in the figure indicate the significance of differences under straw return (S) treatments, while those below the lines indicate the significance of differences under phosphorus fertilizer (P) treatments. The asterisk indicates significant difference, *P < 0.05, **P < 0.01, ***P < 0.01 respectively, while ns indicates no significant difference. Error bars represent the mean ± standard deviation (n = 3).

Straw return and rapeseed single-season P application significantly increased rice ATPase activity (**Figure 2**). Except in 2023, when P fertilizer management had no significant effect on rice ATPase activity 21 days after heading, both straw return and P fertilizer management exerted significant effects on rice ATPase activity after the heading stage. (*P* < 0.05) The interaction between straw return and P fertilizer management significantly affected rice ATPase activity only 14 days after heading. Under the same P fertilizer treatment, straw return significantly increased rice ATPase activity after heading. The ATPase activity of rice under S2 was increased by 5.14–142.57%, 0.69–96.22%, and 8.71–69.30% compared to that under S1 for the three P fertilizer treatments. Under the same straw return treatments, the highest ATPase activity was observed in the P3 treatment at all stages after heading, which was 1.83–156% higher than that in the other treatments.

### 3.3 Plant P accumulation and P utilization

**Figure 3:**
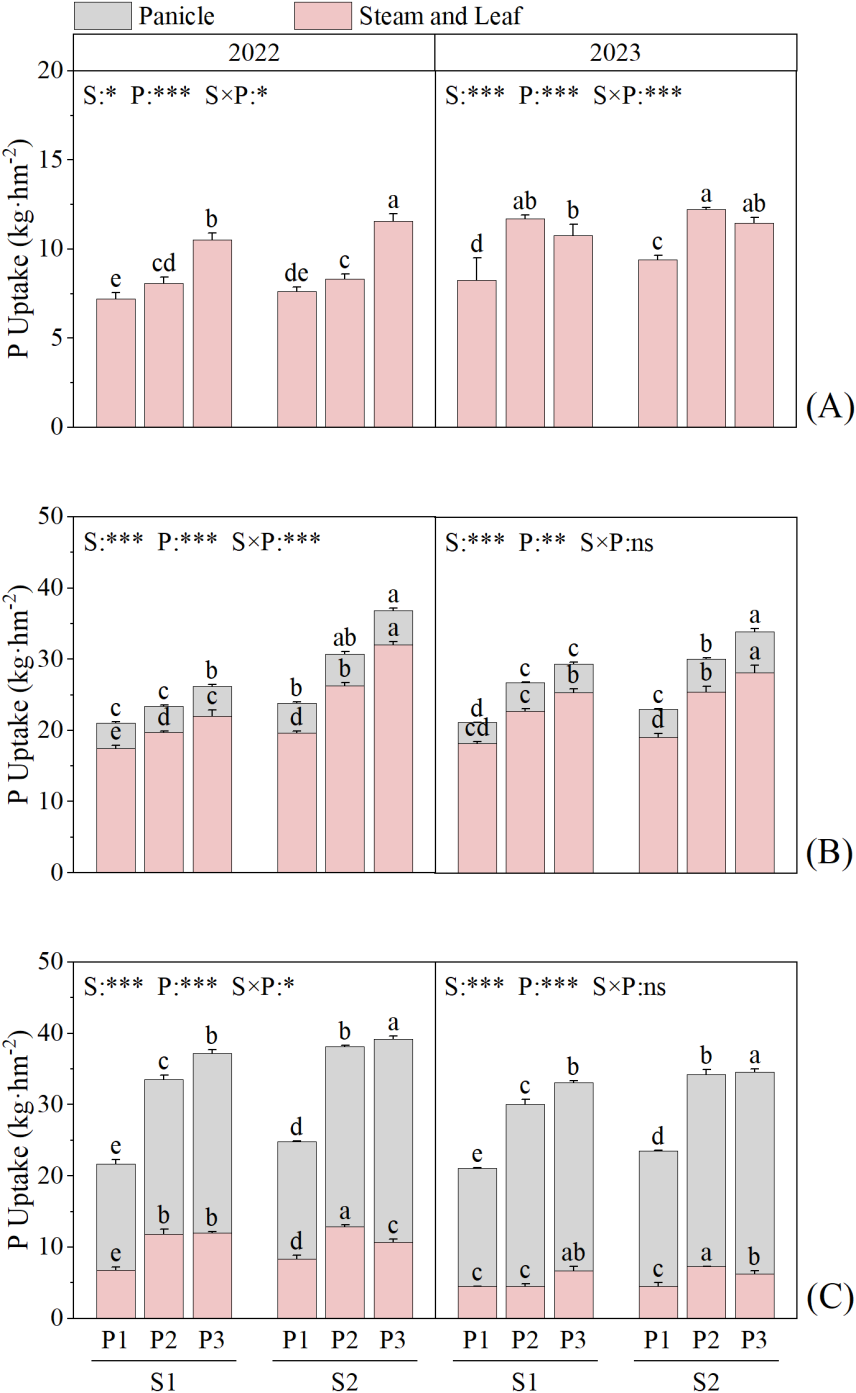
Effects of straw return and phosphate fertilizer treatment on phosphorus uptake of rice in 2022-2023. **(A)** the maximum tillering stage, **(B)** full heading stage, **(C)** maturity stage, the results of ANOVA between different treatments in the lowercase alphabet. S, P, S × P are the interaction between straw return treatment, phosphate fertilizer treatment, straw return and phosphate fertilizer treatment, respectively. The asterisk indicates significant difference, *P < 0.05, **P < 0.01, ***P < 0.001; ns indicates no significant difference. The error bars represent the ± standard deviation of the mean (n = 3).

In the two-year field experiment, straw return and P fertilizer treatments had significant effects on P uptake in rice at each main growth stage (*P* < 0.05) (**Figure 3**). Under all P fertilizer treatments, the P uptake in rice at each main growth stage was higher under S2 than S1, with increases of 2.86–14.17%, 4.51–40.48%, and 3.63–14.64% at different stages. P3 treatment increased the P uptake in rice at heading and maturity stages. Except for the maximum tillering stage in 2023, under the same straw treatments, the P uptake in rice was the highest in the P3 treatment at all stages, which was 9.9–54.3% and 0.96–71.9% higher than that in the other treatments at the heading and maturity stages, respectively.

**Figure 4:**
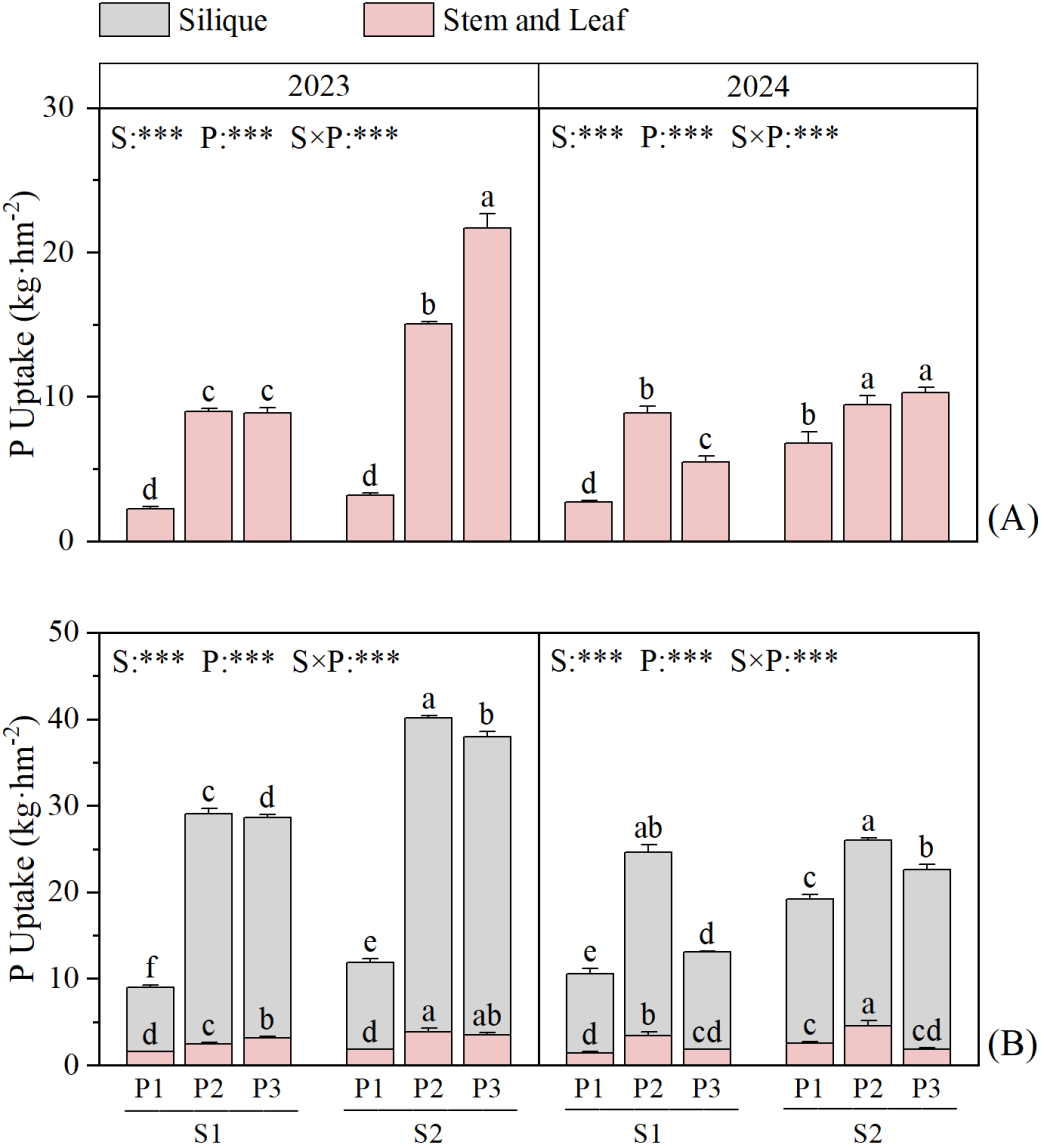
Effects of straw return and phosphate fertilizer treatment on phosphorus uptake of rapeseed in 2023 ~ 2024. **(A)** bolting and budding stage, **(B)** maturity stage, the results of ANOVA between different treatments in the lowercase alphabet. S, P, S × P are the interaction between straw return treatment, phosphate fertilizer treatment, straw return and phosphate fertilizer treatment, respectively. The asterisk indicates significant difference, ***P < 0.001. The error bars represent the ± standard deviation of the mean (n = 3).

S, P, S×P effects on rapeseed P uptake at each stage (*P* < 0.05) (**Figure 4**). Under the same P fertilizer treatment, the P uptake in rapeseed was higher under S2 than S1 at all stages, with increases of 31.75–149.4%, 3.79–67.49%, and 32.45–144.82% under the different P fertilizer treatments. Under S1, at the rapeseed bolting stage, the P uptake showed a trend of P2 > P3 > P1, with that in P2 being 1.44–301.56% higher than that in the other treatments. Under S2, the trend was P3 > P2 > P1, with P uptake in P3 being 8.75–578.17% higher than that in the other treatments. At the rapeseed maturity stage, the P uptake showed a trend of P2 > P3 > P1 under all straw treatments.

In the two-year field experiment, straw return increased the annual P accumulation and partial factor productivity of P fertilizer in the rapeseed-rice rotation (**Table 3**). Under the same P fertilizer treatment, the annual P accumulation and partial factor productivity of P fertilizer under S2 were significantly higher than those in S1 by 9.33–42.56% and 9.72–19.4%, respectively. The recovery efficiency of P fertilizer under S2 increased by 22.5% compared to S1 between 2022 and 2023 but decreased by 24.88% between 2023 and 2024. The agronomic efficiency of P fertilizer under S2 decreased by 8.45% compared to that under S1 between 2022 and 2023 but increased by 67.52% between 2023 and 2024. Under the same straw treatment, the annual P accumulation in rapeseed-rice rotation showed a trend of P2 > P3 > P1. However, the recovery efficiency of P fertilizer showed a trend of P3 > P2, with that in P3 being 9.47–92.51% higher than that in P2. The partial factor productivity of P fertilizer showed a trend of P3 > P2, with that in P3 being 83.04–99.18% higher than that in P2. The agronomic efficiency of P fertilizer showed a trend of P3 > P2, with that in P3 being 145–239.49% higher than that in P2. Compared with P2, P3 increased the recovery efficiency, agronomic efficiency, and partial factor productivity of P fertilizer in rapeseed-rice rotation, thereby increasing the annual yield.

**Table 3:** Effects of straw return and phosphorus fertilizer management on annual phosphorus utilization in rapeseed-rice rotation.

Year	Treatment		Annual P accumulation (kg·hm^-2^)	P fertilizer uptake and utilization efficiency(%)	Partial factor productivity of P fertilizer (kg·kg^-1^)	Agronomic efficiency of P fertilizer (kg·kg^-1^)
2022~2023	S1	P1	30.65e	0	0	0
		P2	62.64c	15.23d	42.31d	8.16b
		P3	65.84b	29.32b	80.54b	20.78a
		Mean	53.04	22.28	61.43	14.47
	S2	P1	36.69d	0	0	0
		P2	78.31a	19.82c	49.51c	7.42b
		P3	77.09a	33.67a	92.8a	19.15a
		Mean	64.03	26.75	71.16	13.29
2023~2024	S1	P1	31.6e	0	0	0
		P2	54.94c	11.12b	52.61d	6.04d
		P3	46.2d	12.17a	96.3b	14.8b
		Mean	44.25	11.65	74.46	10.42
	S2	P1	45.04d	0	0	0
		P2	60.07a	7.16c	57.73c	8.48c
		P3	57.58b	10.45b	114.98a	28.8a
		Mean	54.23	8.81	86.36	18.64
F Value	Y		1271.867**	5819.114**	2029.41**	4.356
	S		1618.305**	18.967**	1191.217**	126.257**
	P		4738.496**	1857.866**	21201.484**	1817.835**
	Y×S		3.681	381.131**	11.975**	225.308**
	Y×P		769.024**	991.733**	239.974**	14.245**
	S×P		3.089	7.158*	221.128**	72.624**
	Y×S×P		100.122**	10.996**	45.922**	98.535**

Different lowercase letters in the same column represent ANOVA results between different treatments in the same year; Y, S and P in the F value are year, straw return treatment and phosphate fertilizer treatment, respectively. Y × S, Y × P, S × P and Y × S × P are the interaction between year, straw return treatment and phosphate fertilizer treatment, respectively. The asterisk indicates significant difference, *P < 0.05, **P < 0.01.

### 3.4 Soil organic matter and phosphorus content

**Figure 5:**
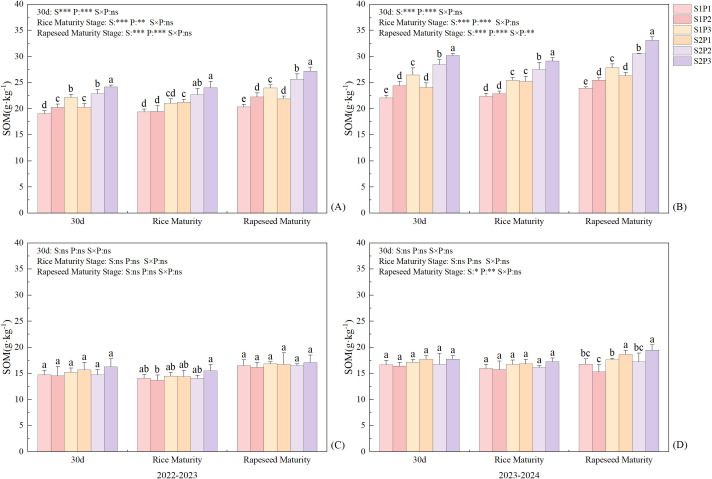
Effects of straw return and phosphorus fertilizer treatments on soil organic matter content from 2022 to 2024, **(A, C)** 0–15 cm, **(B, D)** 16–30 cm. Different lowercase letters in the same group in the figure indicate the ANOVA results among different treatment. S, P, S × P are the interaction between straw return treatment, phosphate fertilizer treatment, straw return and phosphate fertilizer treatment, respectively. The asterisk indicates significant difference, *P < 0.05, **P < 0.01***P < 0.001 respectively; ns indicates no significant difference. Error bars represent the mean ± standard deviation (n = 3).

Straw return and single-season P application to rapeseed significantly increased the S1 soil layer (0–15 cm) SOM content (*P* < 0.01) (**Figure 5**). Under the same P fertilizer treatment, the SOM content in all soil layers was higher under S2 than S1. In the S1 soil layer, the SOM content under S2 was 6.49–12.7%, 12.77–20.24%, and 9.26–18.7% higher than that under S1 in the three P fertilizer treatments. In the S2 (16–30 cm) soil layer, the SOM content under S2 was 1.19–11.23%, 1.35–12.38%, and 1.42–10.18% higher than that under S1 in the three P fertilizer treatments. Under the same straw return treatment, the SOM content in all soil layers was the highest in the P3 treatment. In the S1 soil layer, the SOM content showed a trend of P3 > P2 > P1 in all P fertilizer treatments, with that in P3 being 21.17% and 9% higher than that in P1 and P2, respectively. In the S2 soil layer, the SOM content showed a trend of P3 > P1 > P2, with that in P3 being 4.75% and 14.14% higher than that in P1 and P2, respectively. The content of SOM in S1 soil layer gradually accumulated with time, and there was no obvious accumulation in S2.

**Figure 6:**
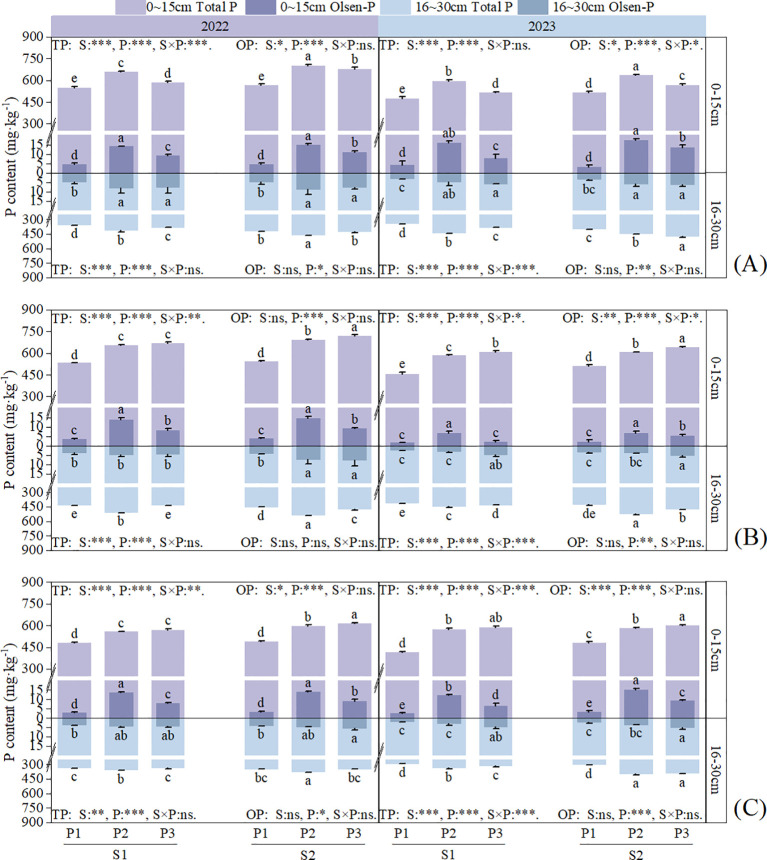
Effects of straw return and phosphorus fertilizer treatments on soil total P (TP) and Olsen-P (OP) content of rice. **(A)** the maximum tillering stage, **(B)** full heading stage, **(C)** maturity stage. Different lowercase letters in the same group in the figure indicate the ANOVA results among different treatment. S, P, S × P are the interaction between straw return treatment, phosphate fertilizer treatment, straw return and phosphate fertilizer treatment, respectively. The asterisk indicates significant difference, *P < 0.05, **P < 0.01, ***P < 0.001 respectively; ns indicates no significant difference. Error bars represent the mean ± standard deviation (n = 3).

**Figure 7:**
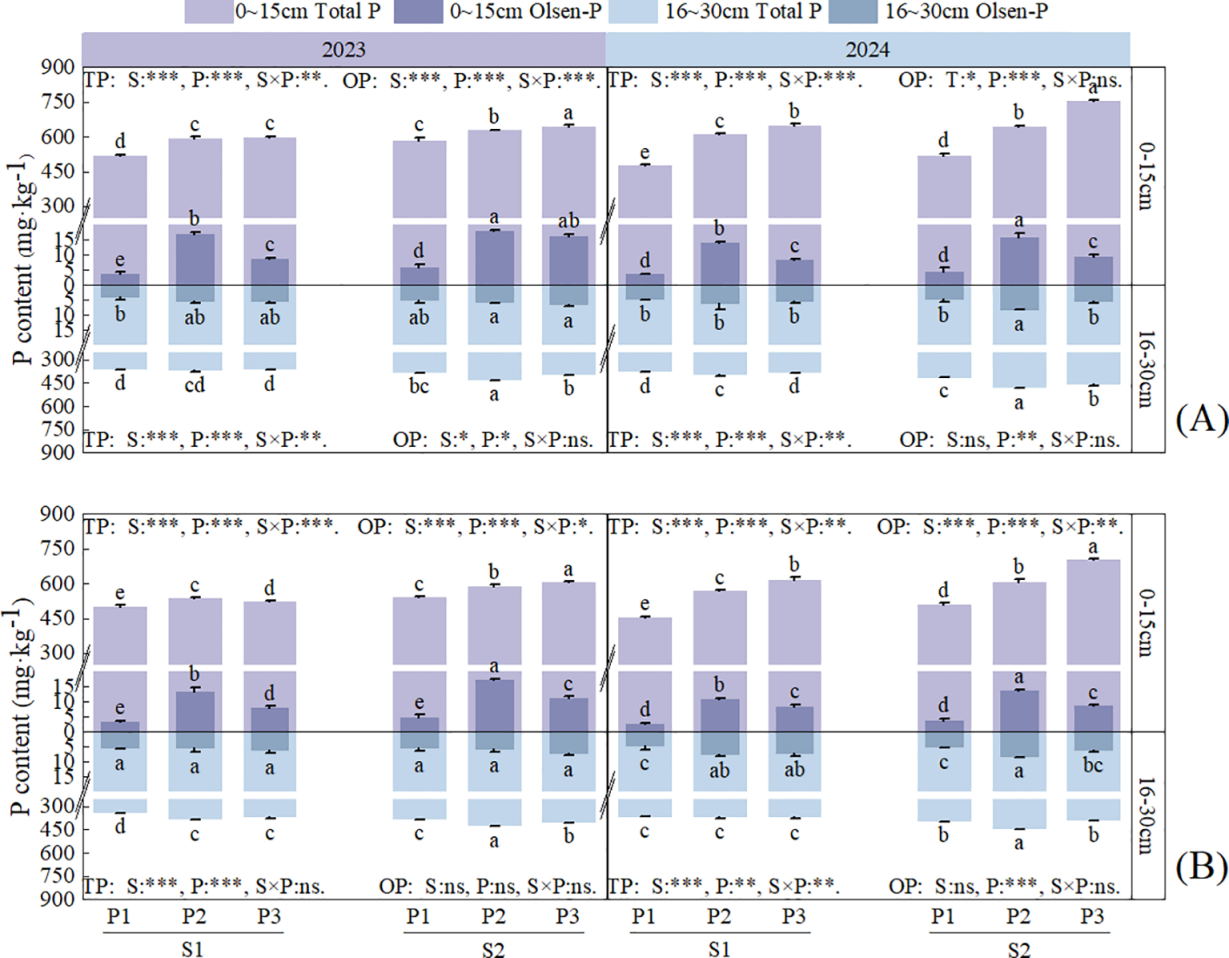
Effects of straw return and phosphorus fertilizer treatments on soil total P (TP) and Olsen-P (OP) content of rapeseed. **(A)** bolting and budding stage, **(B)** maturity stage, the results of ANOVA between different treatments in the lowercase alphabet. S, P, S × P are the interaction between straw return treatment, phosphate fertilizer treatment, straw return and phosphate fertilizer treatment, respectively. The asterisk indicates significant difference, *P < 0.05, **P < 0.01, ***P < 0.001 respectively; ns indicates no significant difference. The error bars represent the ± standard deviation of the mean (n = 3).

Straw return and P application had significant (*P* < 0.01) or highly significant (*P* < 0.001) effects on the total P (TP) content in each soil layer at each stage, and significant effects on the Olsen-P (OP) content in S1 soil layer (*P* < 0.05), however, the OP content in S2 soil layer was only affected by the application of P application (**Figures 6**, **7**). In the two-year field experiment, the TP and OP contents in all soil layers were higher under S2 than S1 in all P fertilizer treatments. In the S1 soil layer, the TP and OP contents under S2 were 1.33–16.19% and 0.69–150.92% higher than those under S1, respectively. In the S2 soil layer, the TP and OP contents under S2 were 2.06–25.41% and 1.06–68.92% higher than those under S1, respectively.

Under the same straw return treatment, in the S1 soil layer, the TP content at 30 days after rice transplantation showed a trend of P2 > P3 > P1 with increasing P application, whereas at the other stages, it showed a trend of P3 > P2 > P1. The TP content in P3 was 4.62–45% and 0.35–16.82% higher than that in P1 and P2, respectively. In the S2 soil layer, the TP content showed a trend of P3 > P2 > P1 at 30 days after transplantation in 2023, whereas at the other stages, it showed a trend of P2 > P3 > P1. In the S1 soil layer, the OP content was the highest in the P2 treatment at all stages and was 207.72–434.5% and 10.14–216.68% higher than that in the P1 and P3 treatments, respectively. In the S2 soil layer, the OP content showed no clear trend but was still higher in the P treatments than in the control treatment.

### 3.5 Soil microbial community structure and phosphorus-soluble bacteria abundance

Straw return and P fertilizer treatments had no significant effect on the composition of soil bacterial communities but did affect the abundance of bacterial communities (**Supplementary Figure 4**). In all treatments, the dominant bacterial phyla in soil (relative abundance > 5%) were *Proteobacteria* (16.19–27.75%), *Acidobacteriota* (18.83–23.63%), *Chloroflexi* (8.77–16.62%), and *Myxococcota* (5.19–9.67%). The relative abundances of *Proteobacteria* and *Acidobacteriota* were highest in the S1 and S2 soil layers, respectively. Compared to the S1 soil layer, the relative abundances of *Proteobacteria*, *Bacteroidota*, and *Desulfobacterota* decreased in the S2 soil layer, whereas the relative abundances of other phyla increased, particularly that of *Acidobacteriota*. In the S1 soil layer, the relative abundance of *Proteobacteria* in the P1 and P2 treatments under S2 increased by 2.86% and 5.95%, respectively, compared to that under S1, whereas the relative abundance of *Acidobacteriota* decreased by 17.67% and 1.35%, respectively. No significant changes were observed in the P3 treatment. Compared with the P1 treatment, the relative abundance of the dominant phyla in the P2 and P3 treatments decreased by 4.08–5.21% and 0.61–4.03%, respectively, among which the relative abundance of *Proteobacteria* decreased by 0.88–3.9% and 7.45–11.26%, respectively.

**Figure 8:**
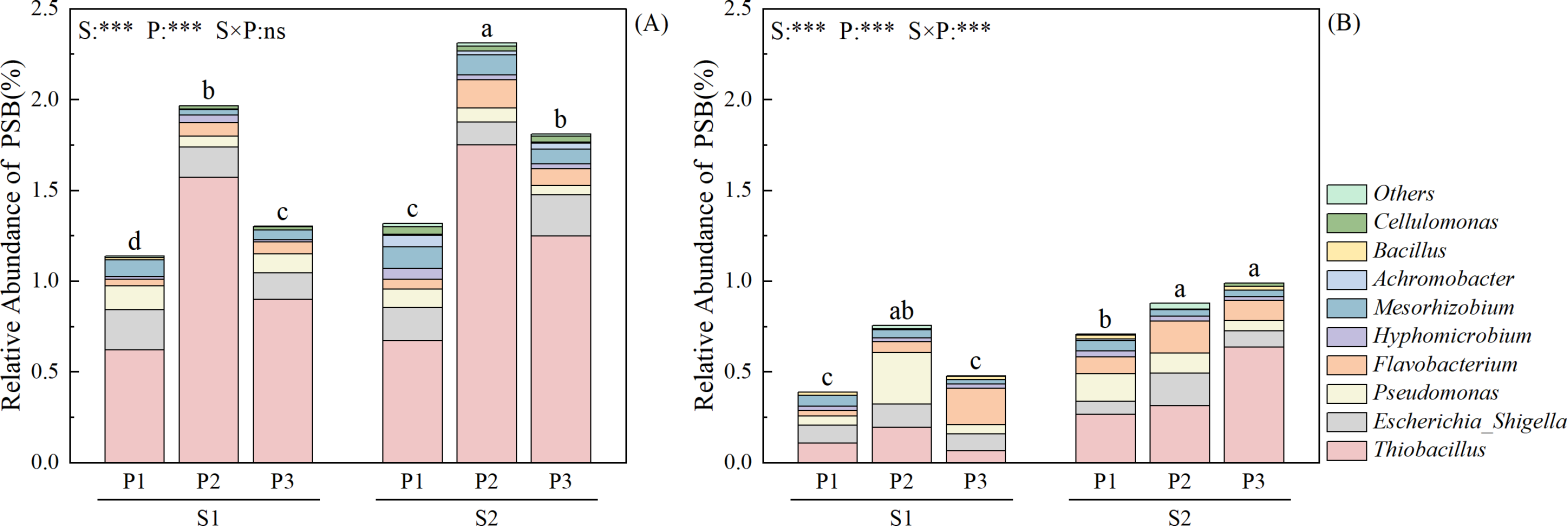
Effects of straw return and phosphorus fertilizer treatments on the relative abundance of phosphorus-soluble bacterial (PSB) genera in soil 30 days after rice transplanting. **(A)** 0–15 cm, **(B)** 16–30 cm, different lowercase letters in the same group in the figure indicate the ANOVA results among different treatment. S, P, S × P are the interaction between straw return treatment, phosphate fertilizer treatment, straw return and phosphate fertilizer treatment, respectively. The asterisk indicates significant difference, ***P < 0.001 respectively; ns indicates no significant difference.

The relative abundance of P-soluble bacteria (RAP) in the soil at 30 days after transplantation varied between the different soil layers (**Figure 8**). In the S1 soil layer, the RAP was higher than that in the S2 soil layer at all stages, with an increase of 82.89–192.72% compared to the S2 soil layer. The RAP in both the S1 and S2 soil layers was higher under S2 than S1 in all P fertilizer treatments, mainly because of the increased relative abundance of *Thiobacillus*, which was 15.85–106.74% higher under S2 than S1. Under the same straw return treatment, the RAP in the S1 soil layer was the highest in the P2 treatment, which was 27.75–75.46% higher than that in the other treatments. In the S2 soil layer, the RAP was the highest in the P2 treatment under S1, which was 75.86% higher than that in the other treatments, and the RAP was highest in the P3 treatment under S2, which was 26.45% higher than that in the other treatments. Straw return and P fertilizer management had highly significant effects on RAP in the S1 soil layer, whereas straw return, P fertilizer management, and their interaction had highly significant effects on RAP in the S2 soil layer (*P* < 0.001).

### 3.6 Relationship between yield, P uptake and soil P

**Figure 9:**
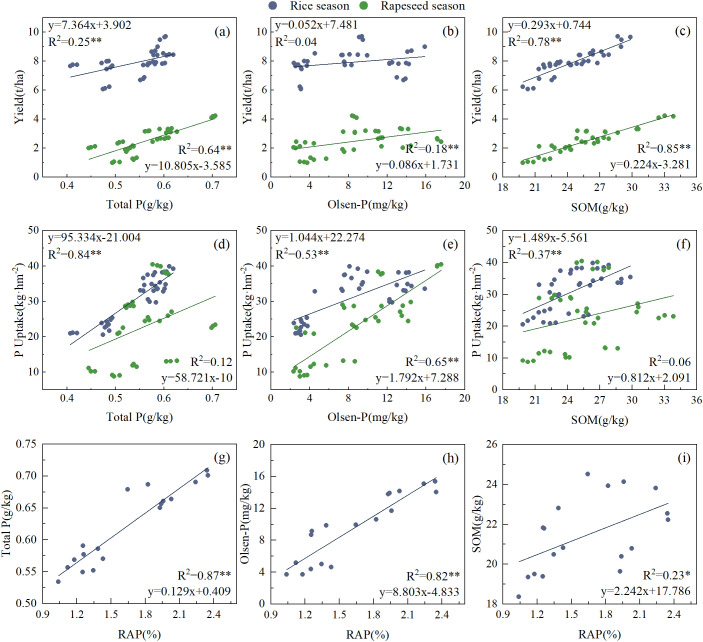
The relationship of yield **(a–c)**, phosphorus absorption **(d–f)**, soil phosphorus content, organic matter and phosphorus-solubilizing microbial abundance **(g–i)** in rice season and rapeseed season. The asterisk indicates *P < 0.05, **P < 0.01, respectively.

Rapeseed yield was significantly correlated with soil TP, Olsen-P, and SOM content (*P* < 0.01), whereas rice yield was significantly correlated with soil TP and SOM content (*P* < 0.01) (**Figures 9a–c**). The results showed that SOM was an important factor affecting rapeseed and rice yields. Rice P uptake was mainly affected by soil TP, OP, and SOM contents, whereas rapeseed P uptake was only affected by soil OP content (**Figures 9d–f**). There was a significant correlation (*P* < 0.05) between soil TP, OP, SOM, and RAP 30 days after rice transplantation, indicating that soil P-soluble bacteria were an important factor affecting soil P availability (**Figures 9g–i**).

## 4 Discussion

### 4.1 Synergistic effect of straw return and phosphorus fertilizer reduction on crop yield

Crop straw contains large amounts of nutrients that promote crop growth and development. Studies have found that crushed straw return with rotary tillage increased the SOM, total nitrogen (TN), TP, and total potassium (TK) in the soil, as well as improved the soil pH, and the numbers of bacteria (Yang et al., 2023), actinomycetes, and fungi in the soil. Moreover, under low N, P, or K conditions, straw return increases crop yield and maintains yield stability (He et al., 2025). Previous studies showed that rapeseed (as the previous crop) can significantly increase the yield of the following crop during a rotation cycle (Chen et al., 2018). Straw return can significantly increase rapeseed and rice yields with a more pronounced increase in rapeseed yield (Wang et al., 2024). The results of this study show that under rapeseed-rice rotation, straw return can increase dry matter accumulation after rice heading and at each main growth stage of rapeseed, and significantly increase the annual yield of rapeseed-rice rotation (**Supplementary Figures 2**, **3**; **Tables 1**, **2**). This may be due to the continuous release of nutrients by straw return, which increases SOM content (**Figure 9c**), thereby enhancing crop uptake and utilization of soil nutrients (Li and Zhong, 2021). And the long-term straw return could synchronize nutrient supply in the soil and the demand of rice plants by regulating the dominant bacterial phyla in the soil, and consequently, increase grain yield of rice (Li et al., 2023).

However, the present study further explored the synergistic effects of straw return and single-season P application on rapeseed. Rapeseed has a high phosphorus demand, whereas soil phosphorus availability is low during the dry season (Zhou et al., 2014), phosphorus supply can significantly promote photosynthate accumulation and transport, thereby enhance crop biomass (Wang et al., 2021b). Previous studies have shown that in the rapeseed-rice rotation system, P fertilizer should mainly be applied during the rapeseed season (Yan et al., 2022), which significantly improves the yield stability of rapeseed but has no significant effect on the overall yield and P accumulation stability of rice (Wang et al., 2025a). The results of this study are consistent with this finding: the enhancement effect of phosphorus application on rapeseed dry matter mass and yield is far stronger than that on rice. Under straw incorporation conditions, the single-season phosphorus application rapeseed treatment (P3) achieved the highest annual dry matter accumulation and annual yield, with significant increases in rice effective panicle number, grains per panicle, as well as rapeseed panicles per plant and grains per panicle. This suggests that when combined with straw incorporation, concentrating phosphorus fertilizer application in the rapeseed season can reduce phosphorus input in the rice season while achieving increased annual yield in the rotation system. This conclusion is supported by existing research: Huo (Huo et al., 2025) found that straw return combined with reduced chemical fertilizer application can significantly increase crop yield and Yan (Yan et al., 2022) showed that excessive P fertilizer input under straw return conditions can lead to crop yield reduction. The underlying mechanism is that in the rapeseed-rice rotation system, phosphorus released through slow decomposition of previous rapeseed straw after incorporation (Yan et al., 2019), combined with improved soil phosphorus availability during the rice season due to alternate flooding and drying patterns (Li et al., 2025), forms a continuous and stable phosphorus supply that perfectly matches the nutrient demand throughout rice’s entire growth period, thereby achieving efficient phosphorus utilization in the rotation system.

### 4.2 Physiological basis for the improvement of phosphorus use efficiency

Phosphorus is a key nutrient for plant growth and development, and improvement in P-use efficiency is of great significance to crop yield and agricultural sustainability (Sun et al., 2021). ATPase provides the critical driving force for the uptake of external H_2_PO_4_^−^ by phosphate transporters (PHT1 family) through hydrolyzing ATP to release energy, thus playing an important role in plant adaptation to low-phosphorus environments (Li et al., 2022). Yang (2002) indicated that when P supply is sufficient (within a certain range of P supply), ATPase activity in plants increases with the increase in P application, which is conducive to P absorption and transport. In this study, the S2P3 treatment maximized the ATPase activity of rice at 14 days after heading (**Figure 2**), which was highly synchronized with the rapid phosphorus uptake period of rice. High ATPase activity indicates a more efficient proton pump (Wang et al., 2021a), which can maintain a high phosphorus uptake rate even at a relatively low soil Olsen-P concentration. This explains why the S2P3 treatment achieved the highest phosphorus uptake when the soil Olsen-P concentration was lower than that in the P2 treatment.

Crop residues tend to increase stable soil organic matter (humic compounds), thereby enhancing the soil’s exchange capacity, facilitating better nutrient retention and slow nutrient release, and thus improving the potential utilization efficiency (Liu et al., 2023). Previous studies showed that straw return can increase available P in soil, thereby promoting the absorption of P by crops (Wang et al., 2020). Combining straw return with a reduction in chemical fertilizer application can effectively ensure that crop yield and nutrient accumulation are maintained or exceed conventional fertilization levels (Huo et al., 2025). Both straw return and P application can increase crop P accumulation, but the effect of straw return on increasing crop P accumulation is higher than that of applying P fertilizer (Xiang et al., 2021). This study showed that straw return and single-season P application to rapeseed significantly increased P accumulation (**Figures 3**, **4**) and fertilizer-use efficiency in the rapeseed-rice rotation system (**Table 3**). Previous studies showed that straw returning to the field can significantly increase the number of soil microorganisms as well as the contents of C, N, and P in microbial biomass (Dong et al., 2026). An increase in the number of soil microorganisms will further promote the secretion of functional substances such as phosphatase (George et al., 2007). Phosphatase, in turn, can accelerate the conversion of organic phosphorus to inorganic phosphorus in the soil, thereby effectively facilitating the absorption and utilization of inorganic phosphorus by crops (Chen et al., 2021). Meanwhile, the high TP content in the P3 treatment (**Figures 6**, **7**) indicates that it has a larger potential phosphorus pool, which can be continuously converted into available phosphorus that is readily absorbable by crops.

### 4.3 The key role of soil-microbe interactions

P exists in various forms in the soil, and the availability of these different forms to crops varies significantly (Luo et al., 2023). Among them, organic P are primarily converted into available P through mineralization for crop uptake, while inorganic P are transformed into soluble inorganic P through microbial dissolution (Zhang et al., 2009). Richardson (Alan and Richard, 2011) showed that soil microorganisms and their interactions play a key role in mediating the distribution of P between the available pool in soil solution and total P, achieved through dissolution and mineralization to form available P, or by immobilizing P into microbial biomass and forming recalcitrant inorganic or organic P forms. P-soluble microorganisms in soil mainly consist of three major groups: P-soluble bacteria, P- soluble fungi, and P-soluble actinomycetes, each group is species-rich and numerous, with current research primarily focusing on phosphate-solubilizing bacteria, among them, *Pseudomonas* and *Bacillus* exhibit strong phosphate-solubilizing abilities (Wakelin et al., 2004). P-soluble bacteria promote the mineralization of organic P by secreting alkaline phosphatase (ALP) (George et al., 2007). Studies have confirmed that ALP plays a major role in soil P (Chen et al., 2021). Soil microbes play key roles in the soil P cycle. Microbial mineralization of soil organic P is driven by the microbial demand for carbon (C), and in low-P soils, microbial biomass is more easily affected by the addition of elements such as C, N, and P (Heuck et al., 2015). Straw return provides soil microbes with a rich source of C, promoting microbial activation and transformation of P, thereby increasing the efficiency of P fertilizer use (Sun et al., 2019). Meanwhile, the paddy-upland rotation system can improve the adverse effects of long-term flooded paddy fields, change the form and effectiveness of soil nutrient elements, and increase the diversity of microbial communities (Zhou et al., 2014). This study found that straw return and P application had no significant effect on the composition of soil bacterial communities, but significantly affected the abundance of bacterial communities (**Supplementary Figure S**). Straw return and P application significantly increased the relative abundance of P-soluble bacteria, in the S1 and S2 soil layer (**Figure 8**). Such changes are primarily dependent on altering the relative abundance of *Thiobacillus*, and research indicates that this bacterial genus can not only function via acid phosphatase activity but also produce sulfuric acid to dissolve phosphate minerals (Etesami et al., 2021).

In addition, the results of this study showed that straw return and P application significantly increased the SOM content (**Figure 5**), further improving the physicochemical properties of the soil and providing a favorable environment for microbial growth and activity (**Figure 9c**). Long-term straw return imports substantial organic matter, which reduces chemical fixation of P through the release of humic acids that complex with Fe/Al oxides (Li et al., 2019). The physical, chemical and biological properties of soil in different seasons of paddy-upland rotation have changed greatly, which will have a great impact on soil phosphorus transformation. In rice season, soil flooding increased aggregate depolymerization, clay deposition, bulk density, available phosphorus release and mobility, while in dry season, soil aggregate formation, porosity increased, water content and available phosphorus mobility decrease (Zhou et al., 2014). At the same time, seasonal alternation of wetting and drying affects soil redox processes and changes soil phosphorus forms and availability (Fan et al., 2008). In the dry season, soil dissolved oxygen content increases, water content decreases, and reduced iron, manganese, etc. are oxidized, and then precipitate with phosphate ions to reduce soil phosphorus availability (Flessa and Fischer, 1992). The anaerobic conditions formed by flooding lead to the decrease of redox potential, the reduction of high valence iron, manganese and other elements to low valence, and the further dissolution of calcium phosphorus, iron phosphorus and other compounds to improve soil phosphorus availability (Li et al., 2025).The slow release of P from straw (Mao, 2015) and the residual effect of P fertilizer from previous crops (Shang et al., 2025) continuously increase the soil available P content (**Figures 6**, **7**). This explains the continuous and effective supply of soil P in the rotation process under the treatment of phosphorus application in rapeseed season combined with straw return(S2P3).

Although we found some significant results, this study had several limitations. The long-term effects of changes in the soil microbial community structure and performance in different soil types requires further research. Future studies should conduct similar experiments in different ecological regions and soil types to verify the universality of the results. Simultaneously, combining molecular biological techniques to study changes in soil microbial community structure and their role in P transformation will provide a more scientific basis for optimizing P fertilizer management.

## 5 Conclusions

In this study, a field experiment was conducted to systematically evaluate the effects of straw return and rapeseed single-season P application on the annual yield, P-use efficiency, soil fertility and microbial community in a rapeseed-rice rotation system. Straw return combined with rapeseed single-season P application (S2P3) treatment significantly increased the dry matter accumulation and annual yield of the rapeseed-rice rotation system, increased the activity of ATPase to increase the uptake and utilization rate of P fertilizer, and increased the total P and Olsen-P contents in the soil through straw decomposition and the activity of soil P-soluble bacteria. It achieved the goal of reducing P and increasing P-use efficiency, provided an efficient and environmentally friendly management model for the sustainable development of the rapeseed-rice rotation system.

## Data Availability

The raw data supporting the conclusions of this article will be made available by the authors, without undue reservation.

## Supplementary material

Supplementary Figure 1 Daily average temperature and precipitation during the experimental period from 2022 to 2024.

Supplementary Figure 2 Effects of straw return and phosphate fertilizer treatment on dry matter accumulation of rice in 2022 **(A, C, E)** and 2023 **(B, D, F)**. **(A, B)** the maximum tillering stage, **(C, D)** full heading stage, **(E, F)** maturity stage, the results of ANOVA between different treatments in the lowercase alphabet. S, P, S × P are the interaction between straw return treatment, phosphate fertilizer treatment, straw return and phosphate fertilizer treatment, respectively. The asterisk indicates significant difference, ***P* < 0.01, ****P* < 0.001; ns indicates no significant difference. The error bars represent the ± standard deviation of the mean (n = 3).

Supplementary Figure 3 Effects of straw return and phosphate fertilizer treatment on dry matter accumulation of rapeseed in 2023 **(A, C)** and 2024 **(B, D)**. **(A, B)** bolting and budding stage, **(C, D)** maturity stage, the results of ANOVA between different treatments in the lowercase alphabet. S, P, S × P are the interaction between straw return treatment, phosphate fertilizer treatment, straw return and phosphate fertilizer treatment, respectively. The asterisk indicates significant difference, **P* < 0.05, ****P* < 0.001. The error bars represent the ± standard deviation of the mean (n = 3).

Supplementary Figure 4 Effects of straw return and phosphorus fertilizer treatments on soil bacterial community structure at the phylum level 30 days after rice transplanting. **(A)** 0–15 cm, **(B)** 16–30 cm.
