# Supplementary figures and images for: Straw return combined with single-season phosphorus application for rapeseed enhances the annual productivity and phosphorus use efficiency under rapeseed–rice rotation
Source: Front Plant Sci. 2026 Mar 17;17:1768595. doi: 10.3389/fpls.2026.1768595 (PMC13035514; doi:10.3389/fpls.2026.1768595)

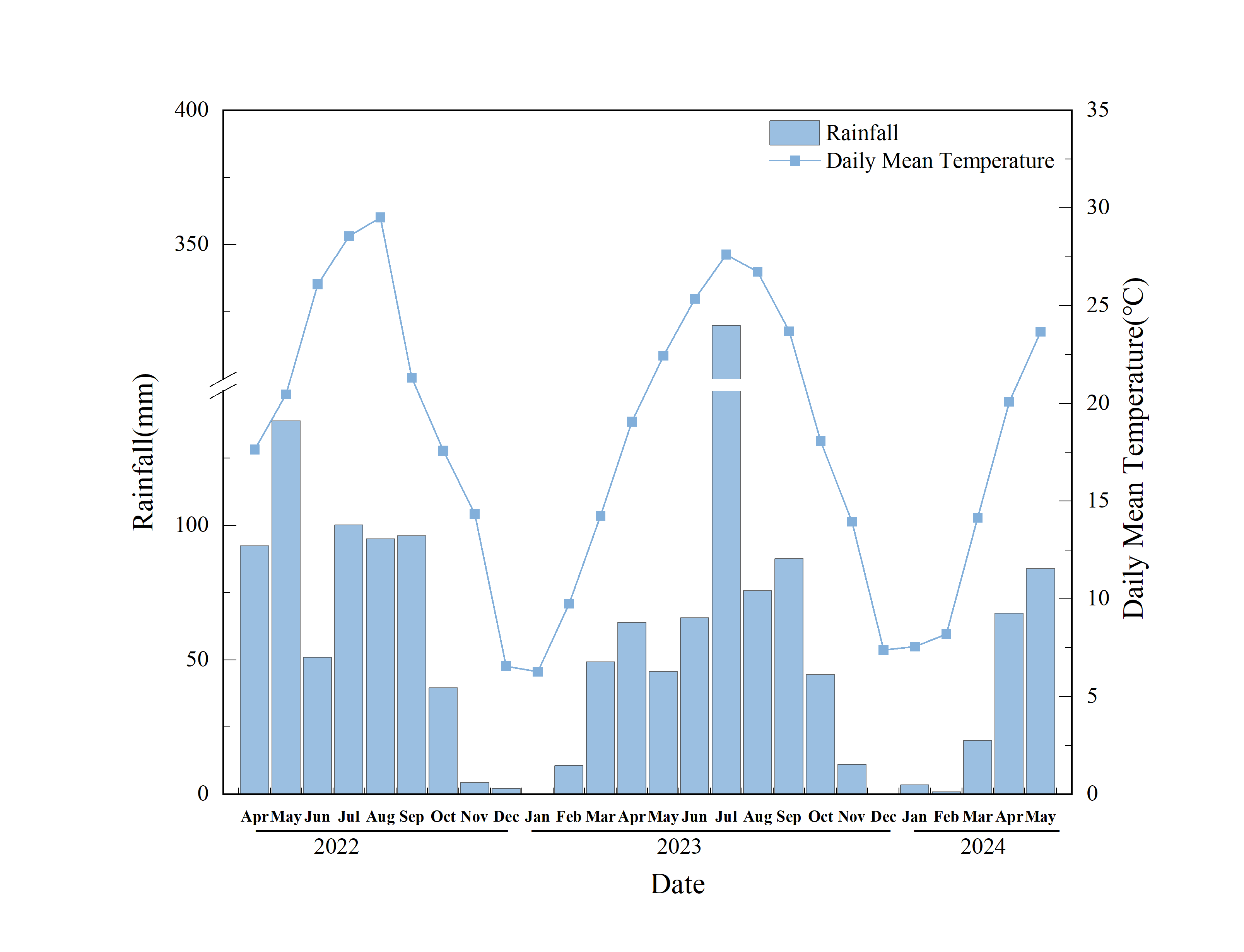

Supplement: Supplementary Figure 1 — Daily average temperature and precipitation during the experimental period from 2022 to 2024. [file Image1.tif]

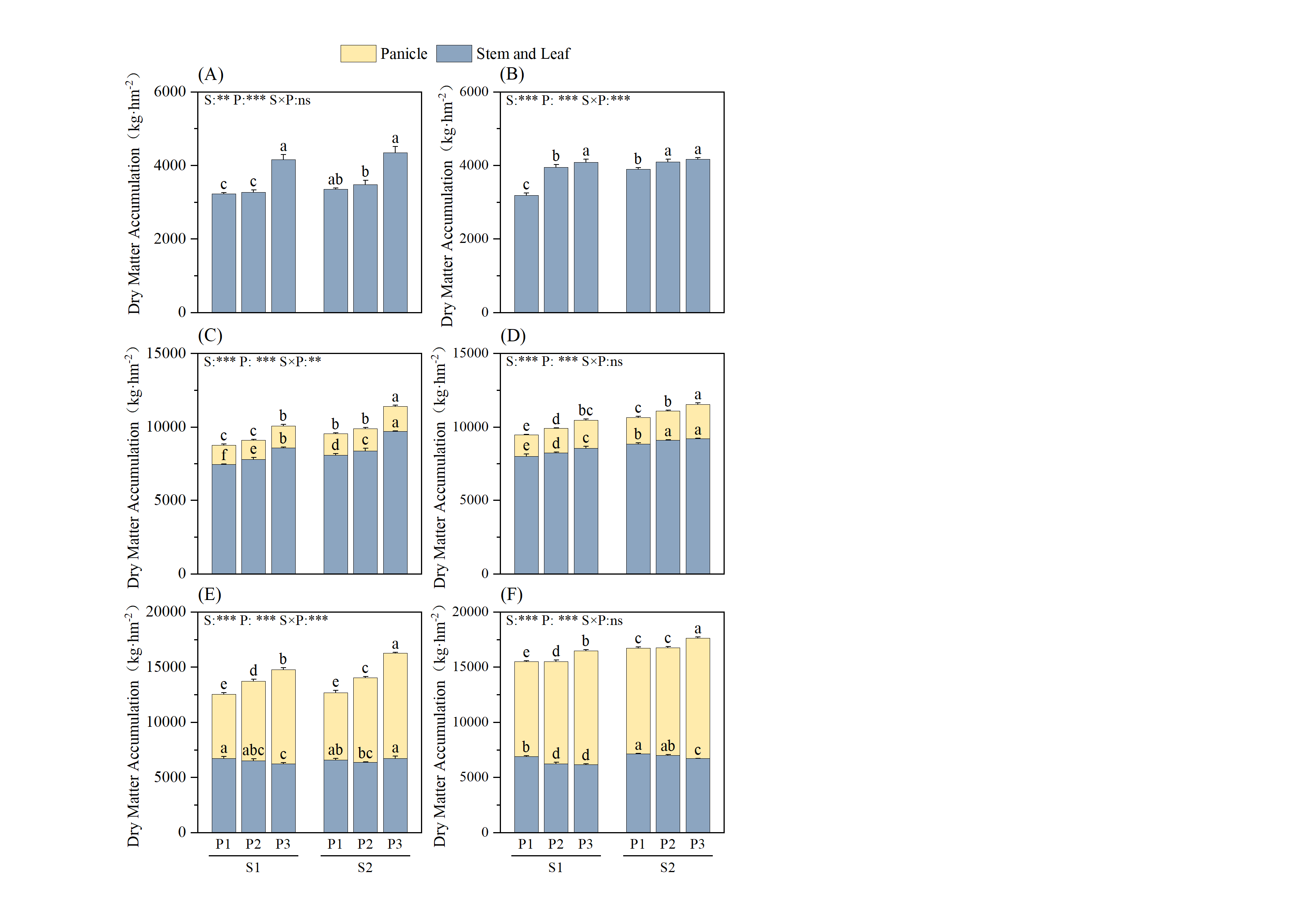

Supplement: Supplementary Figure 2 — Effects of straw return and phosphate fertilizer treatment on dry matter accumulation of rice in 2022 (A, C, E) and 2023 (B, D, F). (A, B) the maximum tillering stage, (C, D) full heading stage, (E, F) maturity stage, the results of ANOVA between different treatments in the lowercase alphabet. S, P, S × P are the interaction between straw return treatment, phosphate fertilizer treatment, straw return and phosphate fertilizer treatment, respectively. The asterisk indicates significant difference, **P < 0.01, ***P < 0.001; ns indicates no significant difference. The error bars represent the ± standard deviation of the mean (n = 3). [file Image2.tif]

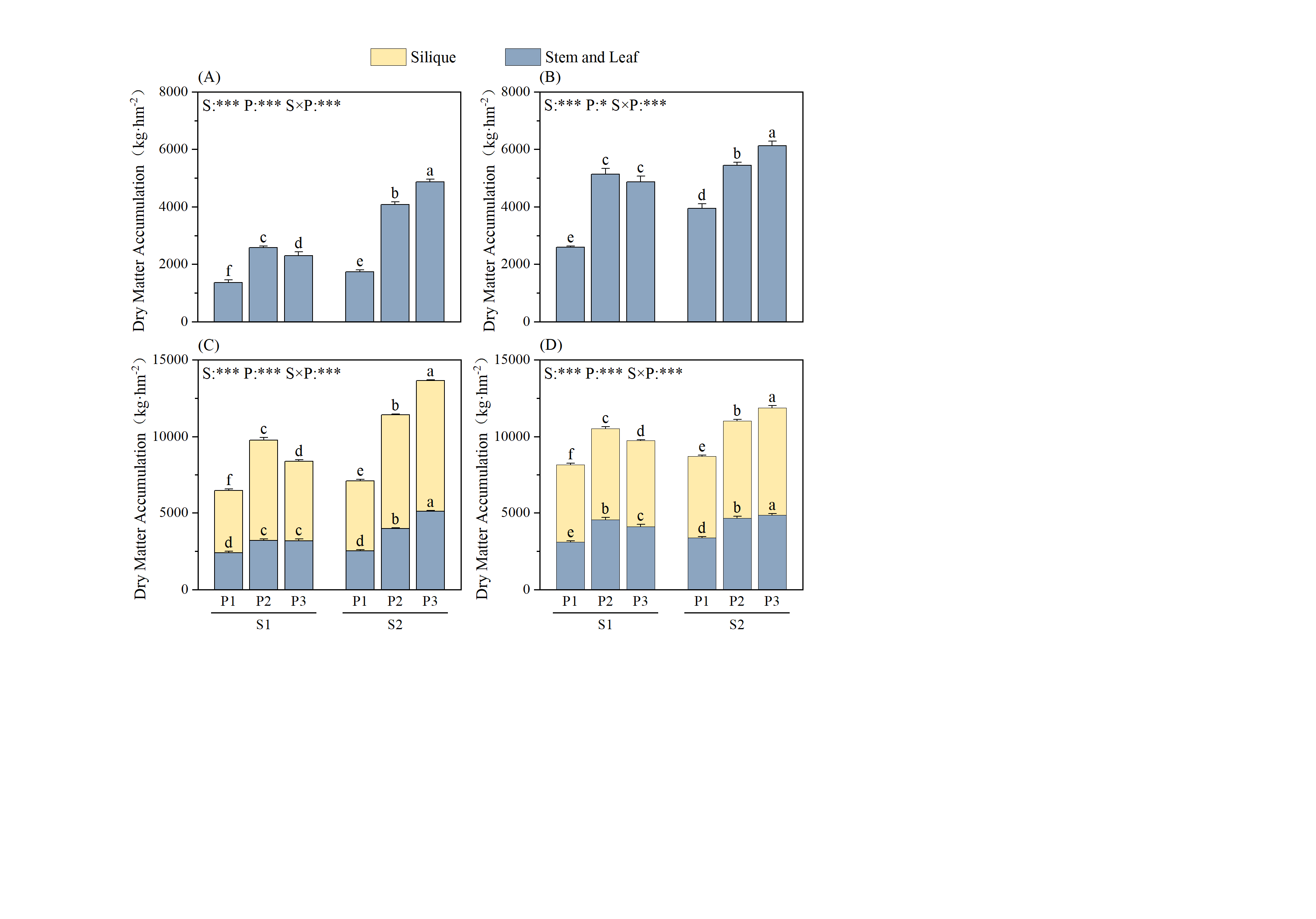

Supplement: Supplementary Figure 3 — Effects of straw return and phosphate fertilizer treatment on dry matter accumulation of rapeseed in 2023 (A, C) and 2024 (B, D). (A, B) bolting and budding stage, (C, D) maturity stage, the results of ANOVA between different treatments in the lowercase alphabet. S, P, S × P are the interaction between straw return treatment, phosphate fertilizer treatment, straw return and phosphate fertilizer treatment, respectively. The asterisk indicates significant difference, *P < 0.05, ***P < 0.001. The error bars represent the ± standard deviation of the mean (n = 3). [file Image3.tif]

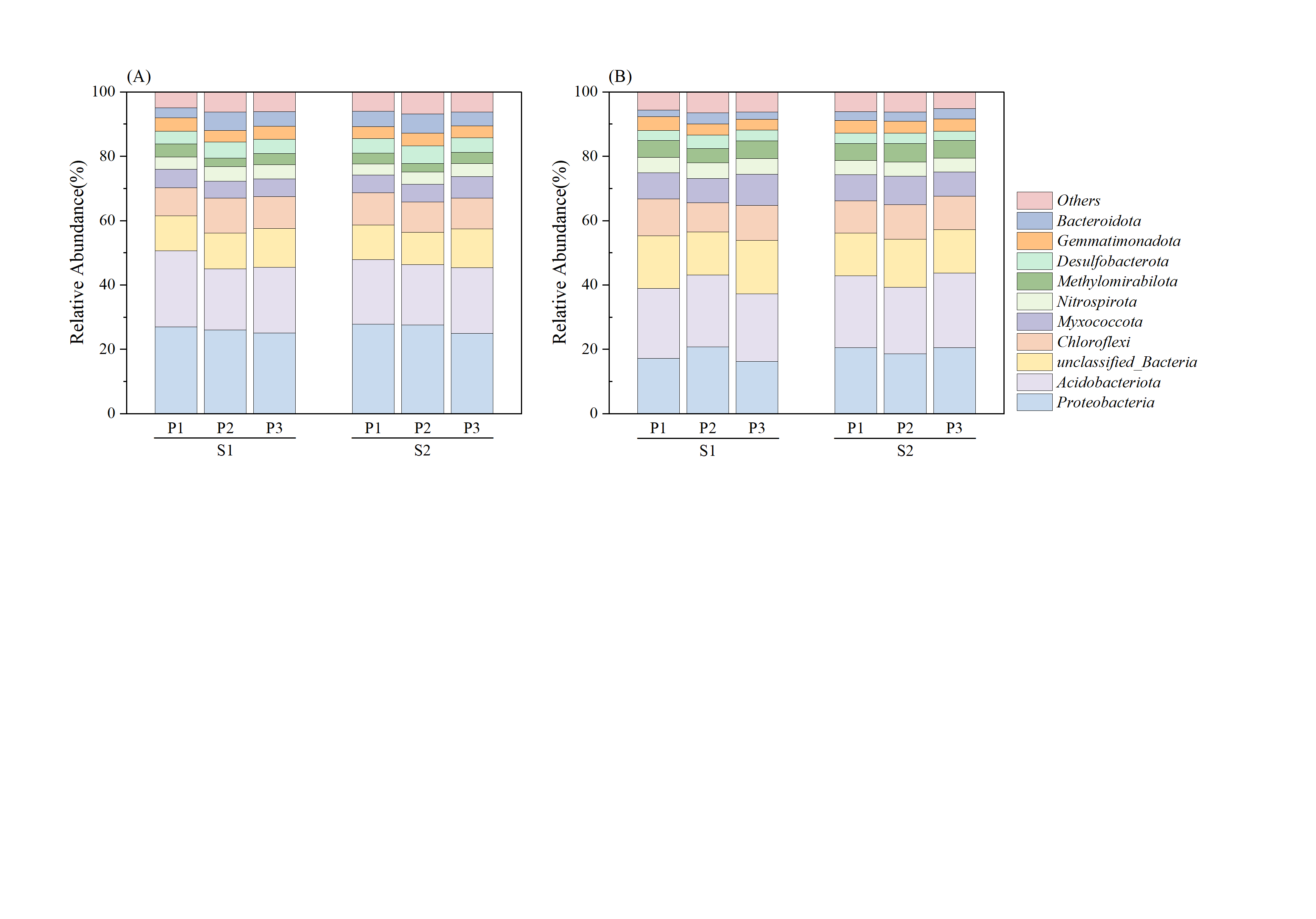

Supplement: Supplementary Figure 4 — Effects of straw return and phosphorus fertilizer treatments on soil bacterial community structure at the phylum level 30 days after rice transplanting. (A) 0–15 cm, (B) 16–30 cm. [file Image4.tif]
